# Supplementary material for: Using a Machine Learning Approach to Predict Snakebite Envenoming Outcomes Among Patients Attending the Snakebite Treatment and Research Hospital in Kaltungo, Northeastern Nigeria
Source: Trop Med Infect Dis. 2025 Apr 11;10(4):103. doi: 10.3390/tropicalmed10040103 (PMC12031592; doi:10.3390/tropicalmed10040103)
Supplement: Supplementary file 1 [file tropicalmed-10-00103-s001.zip › tropicalmed-3465319-supplementary.pdf]

SUPPLEMENTARY APPENDIX

Figure S1. Relationship between age group, sex, and time taken to present to hospital

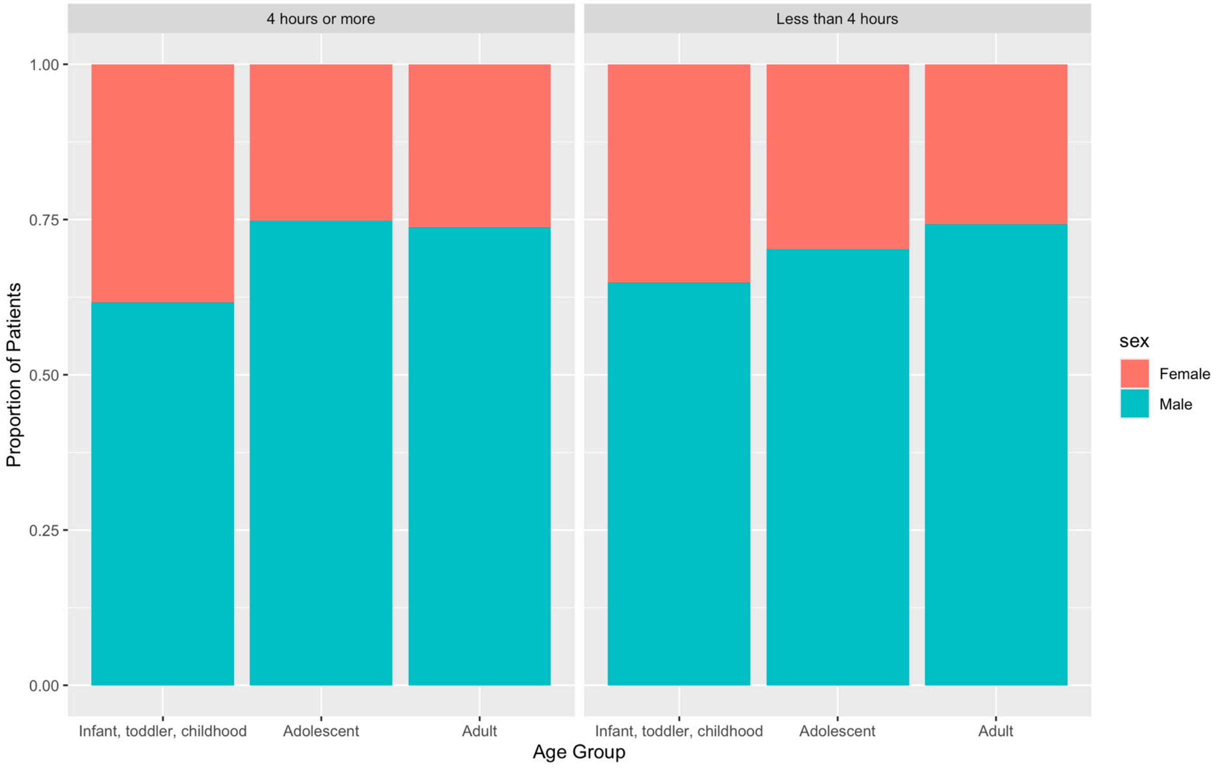

Table S1. Logistic regression for outcome (likelihood of experiencing amputation, debridement or death, compared to recovery), with antivenom variable included

Characteristic	Univariate Models		Multivariable Model	
	**OR**[1]	**95% CI**[1]	**Adjusted OR**[1]	**95% CI**[1]
**Month of Snakebite Occurrence**				
January	—	—	—	—
February	1.04	0.38, 3.01	1.19	0.41, 3.64
March	1.07	0.46, 2.81	1.20	0.48, 3.36
April	0.94	0.41, 2.43	1.05	0.43, 2.88
May	0.52	0.19, 1.49	0.58	0.20, 1.76
June	0.73	0.29, 2.01	0.84	0.31, 2.45
**Age Group**				
Infant, toddler, childhood (0 to 11)	—	—	—	—
Adolescent (12 to 17)	1.86	0.79, 4.73	2.17	0.69, 6.86
Adult (18+)	2.68	1.34, 6.15	3.13	0.86, 12.2
**Sex**				
Female	—	—	—	—
Male	2.52	1.42, 4.81	1.90	0.83, 4.85
**State or Country of Origin**				
Adamawa	—	—	—	—
Bauchi	1.67	0.79, 3.51	1.67	0.76, 3.68
Borno	1.07	0.39, 2.66	0.97	0.34, 2.52

Gombe	0.58	0.32, 1.11	0.82	0.38, 1.79
Other	0.00	0.00, Inf	0.00	0.00, Inf
Taraba	0.59	0.27, 1.27	0.51	0.22, 1.13
Yobe	1.22	0.33, 3.66	0.94	0.24, 2.96
**Occupation**				
Business	—	—	—	—
Civil Servant	0.00	0.00, Inf	0.00	0.00, Inf
Farmer	1.58	0.55, 6.72	1.21	0.39, 5.31
House Wife	0.61	0.17, 2.90	0.68	0.14, 3.95
Other	0.00	0.00, Inf	0.00	0.00, Inf
Student	0.77	0.21, 3.60	0.92	0.23, 4.69
Under Care	0.65	0.19, 2.96	1.35	0.26, 8.36
**Site of Snakebite**				
Lower Limb	—	—	—	—
Other	0.00	0.00, Inf	0.00	0.00, Inf
Upper Limb	1.07	0.65, 1.87	1.14	0.66, 2.05
**Snake Species**				
Carpet Viper (Echis romani)	—	—	—	—
Cobra (Naja)	0.00	0.00, Inf	0.00	0.00, Inf
Mole Viper (Atractaspididae)	0.00	0.00, Inf	0.00	0.00, 0.00
Night Adder (Causus rhombeatus)	0.00	0.00, Inf	0.00	0.00, Inf
Other	0.00	0.00, Inf	0.00	0.00, Inf
Unidentifiable	0.99	0.55, 1.68	1.16	0.63, 2.04
**Antivenom Dose (Number of Vials)**				
0	—	—	—	—
1	2.61	1.04, 8.76	2.34	0.90, 8.03
2 or more	7.22	2.82, 24.5	6.64	2.47, 23.2
**Hours Between Bite and Hospitalization**				
4 hours or more	—	—	—	—
Less than 4 hours	0.50	0.30, 0.80	0.61	0.31, 1.22

[1] OR = Odds Ratio, CI = Confidence Interval

Table S2. Machine learning model results, with antivenom variable included

Features	Model	Sensitivity	Specificity	Positive Predictive Value	Negative Predictive Value	AUROC
**Full Set**	XGBoost	0.74	0.16	0.93	0.04	0.447
	Random Forest	0.68	0.47	0.95	0.09	0.578
	Logistic Regression	0.00	1.00	NaN	0.06	0.500
**Simplified (Four Features)**	XGBoost	0.53	0.53	0.94	0.07	0.529
	Random Forest	0.08	0.95	0.95	0.07	0.512
	Logistic Regression	0.53	0.53	0.94	0.07	0.529
